# Long‐term Effects of 222‐nm ultraviolet radiation C Sterilizing Lamps on Mice Susceptible to Ultraviolet Radiation

**DOI:** 10.1111/php.13269

**Published:** 2020-05-31

**Authors:** Nozomi Yamano, Makoto Kunisada, Sachiko Kaidzu, Kazunobu Sugihara, Aiko Nishiaki‐Sawada, Hiroyuki Ohashi, Ai Yoshioka, Tatsushi Igarashi, Akihiro Ohira, Masaki Tanito, Chikako Nishigori

**Affiliations:** ^1^ Division of Dermatology Department of Internal Related Graduate School of Medicine Kobe University Kobe Japan; ^2^ Department of Ophthalmology Faculty of Medicine Shimane University Izumo Japan; ^3^ Ushio Inc. Tokyo Japan; ^4^ Department of Ophthalmology and Visual Science Graduate School of Biomedical Sciences Nagasaki University Nagasaki Japan

## Abstract

Germicidal lamps that emit primarily 254 nm ultraviolet radiation (UV) are routinely utilized for surface sterilization but cannot be used for human skin because they cause genotoxicity. As an alternative, 222‐nm UVC has been reported to exert sterilizing ability comparable to that of 254‐nm UVC without producing cyclobutane pyrimidine dimers (CPDs), the major DNA lesions caused by UV. However, there has been no clear evidence for safety in chronic exposure to skin, particularly with respect to carcinogenesis. We therefore investigated the long‐term effects of 222‐nm UVC on skin using a highly photocarcinogenic phenotype mice that lack xeroderma pigmentosum complementation group A (*Xpa*‐) gene, which is involved in repairing of CPDs. CPDs formation was recognized only uppermost layer of epidermis even with high dose of 222‐nm UVC exposure. No tumors were observed in *Xpa*‐knockout mice and wild‐type mice by repetitive irradiation with 222‐nm UVC, using a protocol which had shown to produce tumor in *Xpa*‐knockout mice irradiated with broad‐band UVB. Furthermore, erythema and ear swelling were not observed in both genotype mice following 222‐nm UVC exposure. Our data suggest that 222‐nm UVC lamps can be safely used for sterilizing human skin as far as the perspective of skin cancer development.

## Graphical abstract

222‐nm UVC have been reported to exert sterilizing ability comparable to that of 254‐nm UVC without producing cyclobutane pyrimidine dimers (CPDs), the major DNA lesions caused by UV. We investigated the long‐term effects of 222‐nm UVC on skin using *Xpa*‐knockout mice, a highly photocarcinogenic phenotype mice. CPDs formation was recognized only in uppermost layer of epidermis with 222‐nm UVC exposure. No tumors were observed in *Xpa*‐knockout mice and wild‐type mice by repetitive irradiation with 222‐nm UVC. These data reveal that 222‐nm UVC lamps can be safely used for sterilizing human skin as far as the perspective of nonmelanoma skin cancer development.

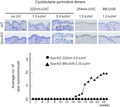

## Introduction

Ultraviolet radiation C (UVC) is defined as 100–280 nm wavelengths UV. UVC from solar UV cannot reach the surface of the earth, because this range of UV is absorbed by ozone layer. Germicidal lamps that primarily emit 254‐nm UVC have been utilized for sterilization because this wavelength is effective for killing bacteria. In spite of the usefulness of 254‐nm UVC lamps for sterilization, it is well known to be harmful to skin and eyes, causing erythema and keratitis, respectively. Its most critical effects on humans and experimental animals are skin carcinogenicity caused by genotoxicity (1, 2, 3, 4). One of the main causes of UV‐induced skin tumors is the formation of covalently linked dimers at dipyrimidine sites of cellular DNA consisting predominantly of cyclobutane pyrimidine dimers (CPDs) and pyrimidine (6‐4) pyrimidone photoproducts (6‐4 PPs) (5). Dipyrimidine photoproducts, in fact essentially deaminated cytosine containing dimeric lesions, are highly mutation‐prone DNA lesions that lead to skin cancer if not repaired (6). Although 254‐nm UVC lamps were not designed to expose human body based on the guidance of the International Electrotechnical Commission for Photobiological Safety of Lamps and Lamp Systems (IEC 62471:2006), lamps emitting much shorter UVC wavelengths (207 and 222 nm) appear to be harmless to murine skin based on the observation that no formation of CPDs was observed (7, 8). These data are fairly convincing because shorter wavelengths do not reach the nuclei of murine epidermal cells (≳10 µm) but do reach bacterial nucleus (≲1 µm), providing similar sterilizing efficacy to 254‐nm UVC germicidal lamps (9, 10, 11, 12). Furthermore, there is evidence that 10 daily exposures of hairless mice to 222‐nm UVC did not produce CPDs, suggesting no carcinogenic consequences with longer‐term irradiation (13). Nevertheless, more direct evidence showing a lack of carcinogenicity of 222‐nm UVC on human skin is required using an animal model known to yield 100% skin‐tumor incidence when wild‐type mice were irradiated with UV, using a broad‐band UVB lamp which emits 280–370 nm wavelength UV, peaking at 312 nm (14, 15). We therefore investigated the photocarcinogenic effects of 222‐nm UVC by repetitive and long‐term irradiation of hairless mice with the 222‐nm UVC. In addition, we also utilized an animal model of xeroderma pigmentosum group A (XP‐A), *Xpa*‐knockout mice. XP is an autosomal‐recessive, hereditary disorder characterized by multiple and early onset of malignant skin tumors, including basal cell carcinoma, squamous cell carcinoma and melanoma, in sun‐exposed areas because of deficiency in the repair of dipyrimidine photoproducts (16, 17). XP patients have a more than 10 000‐fold increased risk of nonmelanoma skin cancer and a more than 2000‐fold increased risk of melanoma before the age of 20 years (18). Among XP clinical subtypes, patients with XP‐A present with the most severe phenotype. Likewise, *Xpa*‐knockout mice are also extremely hypersensitive to UV and highly susceptible to UV‐induced skin carcinogenesis (19, 20, 21, 22).

## Materials and Methods

### UVC source

**Figure 1:**
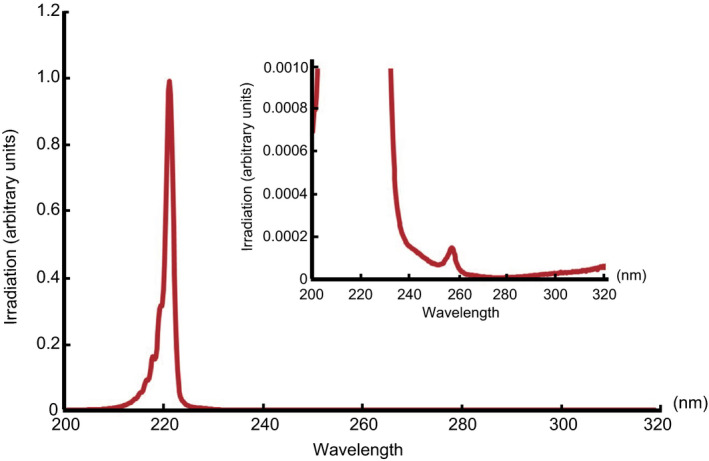
Measured spectra of irradiator A. UVC region, the integrating intensity of 230–235 nm wavelengths UV is 0.26% of the integrating intensity of 200–230 nm wavelengths UV, and the integrating intensity of 235–280 nm wavelengths UV is 0.13% of the integrating intensity of 200–230 nm wavelengths UV. UVB region, the integrating intensity of 280–320 nm wavelengths UV is 0.04% of the integrating intensity of 200–230 nm wavelengths UV. The 222‐nm UVC lamp is designated as irradiator A. [Correction added on July 08, 2020, after first online publication: The figure was updated.]

A krypton‐chloride (Kr‐Cl) excimer lamp and an optical filter that restricted emission to 200–230 nm wavelengths UV, with maximum output wavelength, was 222 nm and full‐width‐half‐maximum of 2 nm. This assembly is called SafeZone UVCⓇ (Ushio Inc. Tokyo, Japan), which is in the trademark registration process. The lamp unit is composed of a lamp, air‐cooling fan, mirrors and a custom band‐pass filter (irradiator A). The installed filter was used to remove nearly all but the dominant 222 nm emission wavelength (Fig. 1). A second 222‐nm UVC lamp unit, irradiator B, was constructed using a stack of three filters that have similar characteristics to the irradiator A filter. The irradiation amount of irradiator B at the wavelength of 235 to 280 nm is <1% of irradiator A (Fig. 1). The intensity of 222 nm light was measured using an S‐172/UIT250 accumulated UV meter (Ushio Inc.) and was found to be 1 mW cm^−2^ at 300 mm from the emission window. For 254‐nm UVC lamp, the irradiator was a low‐pressure mercury lamp (FL‐4W × 1; AS ONE, Osaka, Japan). Before the start of exposure, irradiance was measured with UVD‐S254/ UIT‐250 (Ushio Inc.). The radiation intensity at 11 cm from the irradiation window was 400 μW cm^−2^.

### UVB source

A bank of six TL 20W/12RS fluorescent lamps (Philips, Eindhoven, Holland) was used to irradiate the mice. These lamps emit a continuous spectrum from 275 to 390 nm, with a peak at 313 nm; 65% of that radiation is within the UVB range. The irradiance was 3.8 J m^−2^ s^−1^ at a distance of 40 cm, as measured by an UVR‐305/365D digital radiometer (Tokyo Kogaku Kikai KK, Tokyo, Japan).

### Mice

*Xpa*‐knockout mice with CBA, C57BL/6 and CD‐1 chimeric backgrounds (19) were backcrossed to hairless albino mice from the Balb/cA Kud‐hr inbred strain. The resulting hairless albino *Xpa* +/+ and *Xpa* −/− mice, aged 9–20 weeks, were used. Mice were housed under specific pathogen‐free conditions, and all animal experiments were conducted according to the Guidelines for Animal Experimentation at Kobe University Graduate School of Medicine. For skin tumor production, we followed our published protocol for hairless mice (21). Nine mice (12–13 weeks) of each group were placed 30 cm below the UV source (222‐nm UVC) and irradiated with 5.0 kJ m^−2^ for wild‐type mice three times a week, 0.5 and 1.0 kJ m^−2^ for *Xpa*‐knockout mice twice a week for 10 weeks. Our previous study of *Xpa*‐knockout mice with 0.25 kJ m^−2^ broad‐band UVB once a week was referred as a positive control group for tumor yield (21). After 10 weeks of 222‐nm UVC or UVB exposure, we monitored tumor formation for another 15 weeks. During experiments, mice were sedated using a combination of intraperitoneal administration of pentobarbital and isoflurane inhalation. Observations and measurements of ear swelling were performed under mild sedation by similar method. After 10 weeks UV irradiation followed by 15 weeks observation, mice were sacrificed by cervical dislocation under inhalation anesthesia using isoflurane. When we performed repetitive exposure experiments on mice, we woke them up in a box for a few minutes so that they could move around. For ophthalmological evaluation, the eyes of some of *Xpa*‐knockout mice and wild‐type mice irradiated with 222‐nm UVC were utilized at the end of protocol for skin tumor production and the eyes of *Xpa*‐knockout mice irradiated with 0.5 kJ m^−2^ broad‐band UVB twice a week using the same exposure and observation protocol. All experimental protocols described in Methods were approved by review boards of Institute for Experimental Animals (Approval number: P180207‐R2) and Committee on Genetically Modified Organisms (Approval number: 30‐06), Kobe University Graduate School of Medicine.

### ELISA immunohistochemistry

For detection of serum CXCL1 in either genotype after UV exposure, ELISAs were performed according to the manufacturer’s instructions (R&D Systems) (21).

### Immunohistochemistry

For detection of CPDs in mouse skin, specimens were collected 3 h following UVB irradiation. Skin specimens were fixed in 10% neutralized formalin and embedded in paraffin. Immunohistochemical staining for detection of monoclonal antibody against CPDs (TDM‐2 1:5000 dilution) was performed as described previously (21). Specimens were observed with a Biozero BZ‐X710 microscope (Keyence, Osaka, Japan).

### Statistics

Differences between groups were assessed for significance using Student’s *t*‐test. *P* values <0.05 were considered to be statistically significant.

## Results

### The evaluation of CPDs formation after 222‐nm UVC irradiation

**Figure 2:**
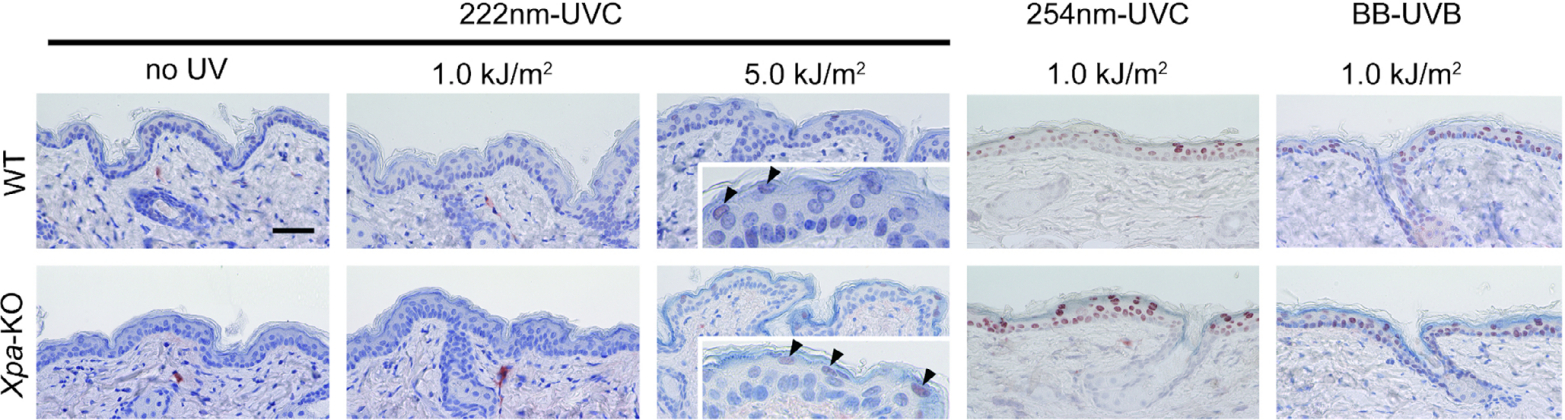
CPDs formation after 222‐nm UVC irradiation. Dorsal skin from *Xpa*‐knockout mice and wild‐type mice was taken 3 h after 222‐nm UVC exposure with 1.0 or 5.0 kJ m^−2^ and subjected to immunohistochemistry with monoclonal antibody against CPDs. Inset photographs were magnified at the epidermis; faintly stained cells are indicated with arrowheads. Scale bar: 50 µm. Strong positive cells for CPDs are shown in each genotype 3 h after either 254‐nm UVC with 1.0 kJ m^−2^ or broad‐band UVB with 1.0 kJ m^−2^.

We utilized 222‐nm UVC lamps, which emit almost exclusively 222‐nm UVC. The lamp unit consists of a lamp, a fan for cooling, mirrors and a custom band‐pass filter. The lamp unit is designated as “irradiator A” in the remainder of this report (Fig. 1). First, we evaluated CPDs formation in the epidermis of hairless albino *Xpa*‐knockout (hereafter *Xpa*‐knockout) and hairless albino wild‐type (hereafter wild‐type) mice after 222‐nm UVC irradiation as previously described in which no CPDs formation had been observed in the dorsal skin of albino hairless mice by a single irradiation with 4.5 kJ m^−2^ of 222‐nm UVC and ten days repetitive irradiation with 4.5 kJ m^−2^ of 222‐nm UVC (13). The strong CPDs‐positive cells were observed after either 254‐nm UVC or broad‐band UVB exposure. When we irradiated mice with 222‐nm UVC at 1.0 kJ m^−2^, no CPDs were detected 3 h after exposure in epidermis of both genotypes of mice. On the other hand, very faint staining for CPDs were recognized in the uppermost part of epidermis, the subcorneal region, after irradiation with 222‐nm UVC at a dose of 5.0 kJ m^−2^ in both genotype of mice (Fig. 2).

### No inflammatory response induced by 222‐nm UVC irradiation

**Figure 3:**
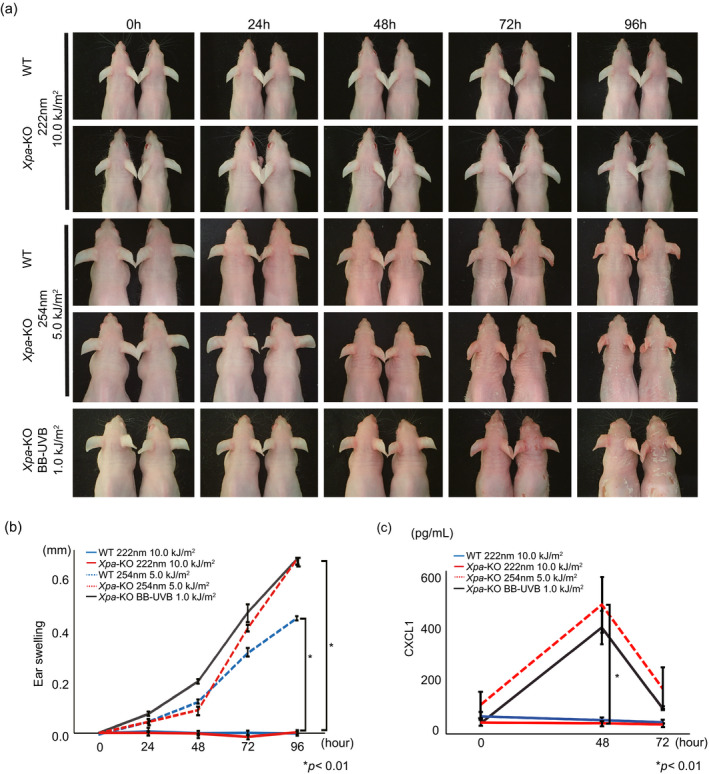
222‐nm UVC does not induce an inflammatory response in mouse skin. (a) Wild‐type and *Xpa*‐knockout mice were irradiated with 222‐nm UVC nm, 254‐nm UVC, and broad‐band UVB and observed for erythema at the indicated time points. Two mice were in each group, and the same pairs were observed and photographed. (b) Ear swelling following 222‐nm UVC nm, 254‐nm UVC and broad‐band UVB irradiation. Error bars represent standard error. *P* value is shown in difference between 254‐nm UVC with 5.0 kJ m^−2^ and 222‐nm UVC with 10.0 kJ m^−2^ of each genotype mice. (c) Serum levels of CXCL1 at 24, 48 and 72 h after 222‐nm UVC nm (wild‐type: *n* = 3, *Xpa*‐knockout: *n* = 8), 254‐nm UVC (*Xpa*‐knockout: *n* = 3) and broad‐band UVB (*Xpa*‐knockout: *n* = 4). Error bars represent standard error. *P* value is shown in difference between 254‐nm UVC with 5.0 kJ m^−2^ and 222‐nm UVC with 10.0 kJ m^−2^ of *Xpa*‐knockout mice.

We have already shown that the inflammatory response after UVB (280–315 nm) irradiation is a strong promoting factor for developing skin cancers (21, 23). Therefore, we studied whether 222‐nm UVC irradiation could induce inflammation in mouse skin. In that sense, we considered *Xpa*‐knockout mice, characterized by a highly photocarcinogenic phenotype with a strong inflammatory response to UV, to be a suitable model to predict human skin tumor formation (21). When the mice were irradiated with 10 kJ m^−2^ of 222‐nm UVC, estimated to be 100 times the sterilizing dose (8), neither erythema nor ear swelling was observed in both genotype mice, while strong erythema and ear swelling was observed on both genotype mice after 254‐nm UVC exposure (Fig. 3a,b). Next, we measured the serum level of CXCL1 after 222‐nm UVC irradiation, since we previously reported CXCL1 as a key inflammatory chemokine and involved in UV‐induced skin carcinogenesis in *Xpa*‐knockout mice (21). The serum level of CXCL1 did not elevate in both genotype mice after a single exposure to 222‐nm UVC at 10 kJ m^−2^, whereas a significant elevation of CXCL1 was observed in *Xpa*‐knockout mice 48 h after either 254‐nm UVC or broad‐band UVB irradiation (Fig. 3c).

### Chronic 222‐nm UVC irradiation did not induce skin tumors

**Figure 4:**
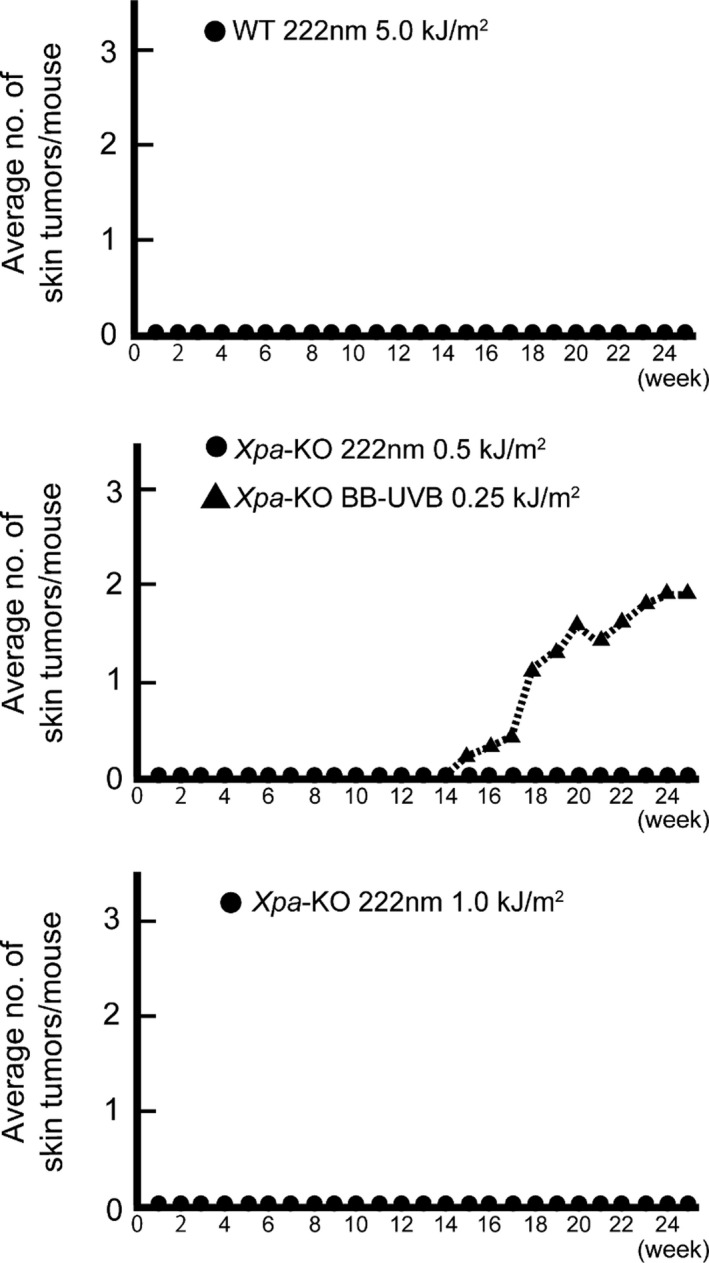
Chronic 222‐nm UVC does not cause skin tumors. Skin tumor production by 222‐nm UVC for both genotype mice. The average number of skin tumors/mouse was plotted. *Xpa*‐knockout mice were irradiated with 0.5 or 1.0 kJ m^−2^ 222‐nm UVC twice a week and 0.25 kJ m^−2^ broad‐band UVB once a week and wild‐type mice were with 5.0 kJ m^−2^ 222 nm three times a week for 10 weeks followed by observation of skin tumor development for 15 weeks. All groups were composed of nine mice. Our previous study of *Xpa*‐knockout mice with 0.25 kJ m^−2^ broad‐band UVB once a week was referred as a positive control group for tumor yield (dotted line) (21).

We utilized a protocol in which 100% of *Xpa*‐knockout mice would develop skin tumors after 10 weeks irradiation with broad‐band UVB followed by 15 weeks of observation (21). Based on an estimated sterilizing dose of 0.1 kJ m^−2^ 222‐nm UVC (8) and a 10 000‐fold greater susceptibility to develop UV‐induced skin tumors in patients with XP in comparison with the general population (18), we set the dose of 222‐nm UVC per exposure for wild‐type with 5.0 kJ m^−2^ and for *Xpa*‐knockout mice with 0.5 and 1.0 kJ m^−2^. We therefore irradiated mice dorsal skin with 222‐nm UVC for 10 weeks, at the dose 0.5 or 1.0 kJ m^−2^ twice per week for *Xpa*‐knockout mice, and at 5.0 kJ m^−2^ three times a week for wild‐type mice. By contrast with our previous study showing an 80% incidence of skin tumors from broad‐band UVB exposure for *Xpa*‐knockout mice (0.25 kJ m^−2^, once a week) (21), we observed no tumors in any of the mice with 222‐nm UVC irradiation, indicating that 222‐nm UVC did not exert photocarcinogenic effects (Fig. 4). Although we speculated that one of the reasons why chronic 222‐nm UVC exposure did not cause photocarcinogenic effects was its shorter wavelength in UV range that could reach only outermost one layer of epidermis and its lower penetrance preventing it from reaching the basal layer, a critical site of induction of skin tumors (24), it remains to be assured for an penetration level of 222‐nm UVC if epidermis is not covered by the stratum corneum or skin barrier is damaged. We observed tumor incidence in both genotypes of male mice (*n* = 2) in the same cage, which resulted in sustained skin injuries by scratching and biting each other, using the same experimental protocol. Still, no tumor formation was observed by long‐term 222‐nm UVC irradiation for both genotype mice, although they presented with a number of significant dorsal skin wounds. These data indicate that the 222‐nm UVC does not exert photocarcinogenicity even when the skin barrier is damaged (see Figure S1).

### No effect of chronic 222‐nm UVC on the mouse eye

**Figure 5:**
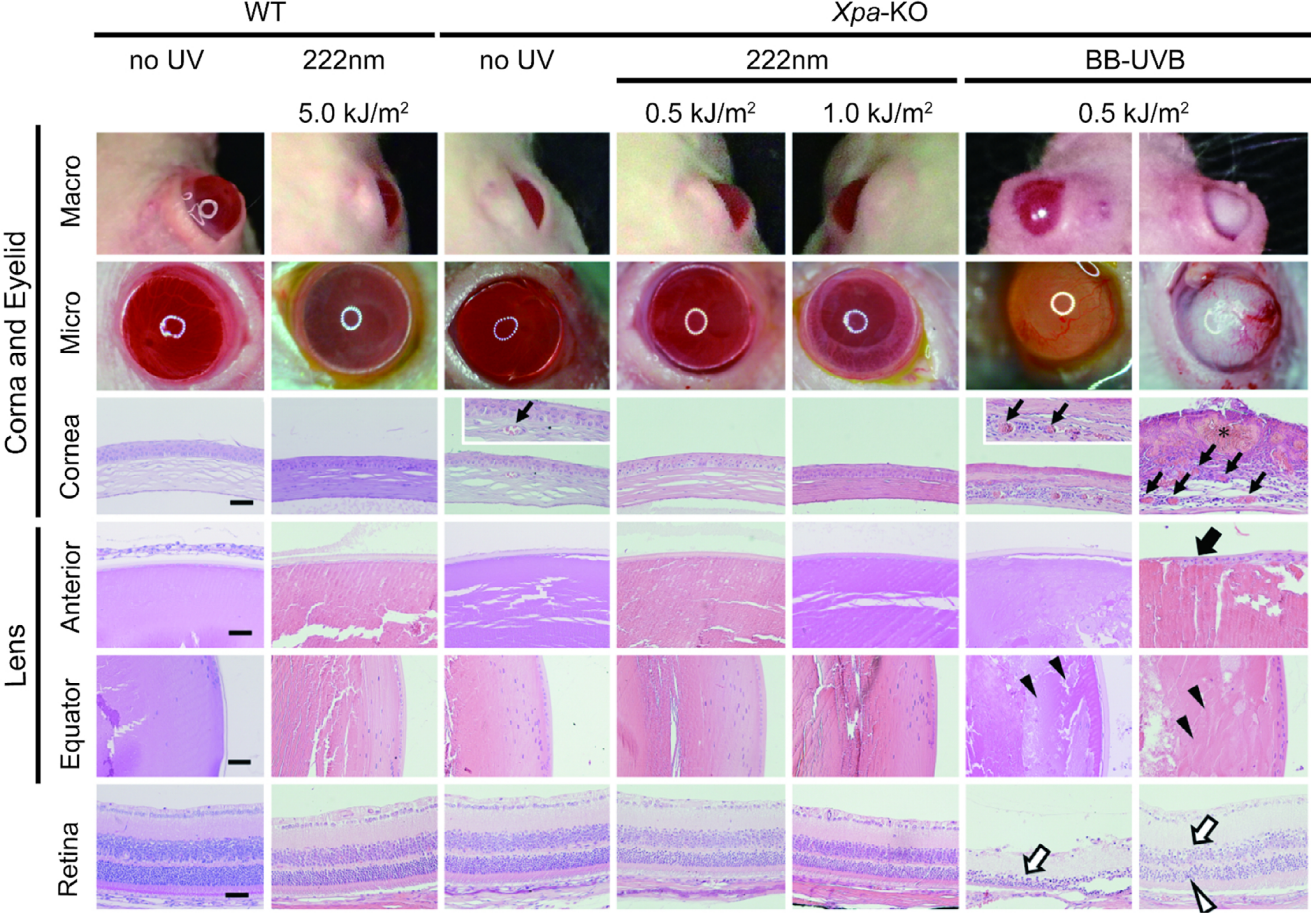
No effect of chronic 222‐nm UVC irradiation on eyes. Ophthalmological evaluations of effects of 222‐nm UVC irradiation. Pathological studies with hematoxylin & eosin stain were shown in cornea, lens (equator and anterior) and retina 15 weeks after the end of chronic exposure experiment by each UV source (Fig. 4). Arrows and asterisks show neovascularization of corneal stroma and ulceration with scarring changes, respectively. Proliferating, stratified lens epithelial cells and disorganized lens cortex were indicated by filled arrow and filled arrow heads, respectively. In retina, thinned inner nuclear layers (closed arrows) and moderately damaged outer nuclear layers (closed arrowheads) were shown. Scale bar: 50 µm.

It was already confirmed that 222‐nm UVC irradiation did not induce acute corneal damage nor reactions in Sprague Dawley rats (25). In this study, we assayed for several chronic ophthalmic effects of 222‐nm UVC exposure, by macroscopic observations and histopathological evaluations (Fig. 5), which was compared with those of broad‐band UVB exposure. In analyses of eyelids and cornea, neovascularization and corneal haze were observed in *Xpa*‐knockout mice irradiated with broad‐band UVB. Histologically, a number of vessels invading and reaching the center of the cornea and abnormal increment cells in the corneal stroma were present. Ulcers with scarring were observed in the corneal epithelium, in the corneal opacification in *Xpa*‐knockout mice irradiated with broad‐band UVB. Cataracts were clearly observed in *Xpa*‐knockout mice irradiated with broad‐band UVB, showing that lens epithelial cells were proliferating and stratified, with disorganization of the lens cortex. Retinal pigmented epithelium and the outer nuclear layer were most sensitive to the UV‐induced damage (26, 27). Thinned inner nuclear layers and moderately damaged outer nuclear layers were also observed in *Xpa*‐knockout mice irradiated with broad‐band UVB. On the other hand, all 222‐nm UVC irradiated mice, irrespective of *Xpa*‐genotype, showed no significant changes on retinal tissue throughout these examinations (Fig. 5), suggesting that 222‐nm UVC was absorbed on the ocular surface (28) and did not reach the lens and retina.

### 222‐nm UVC did not produce dipyrimidine photoproducts and inflammatory responses in mouse skin

**Figure 6:**
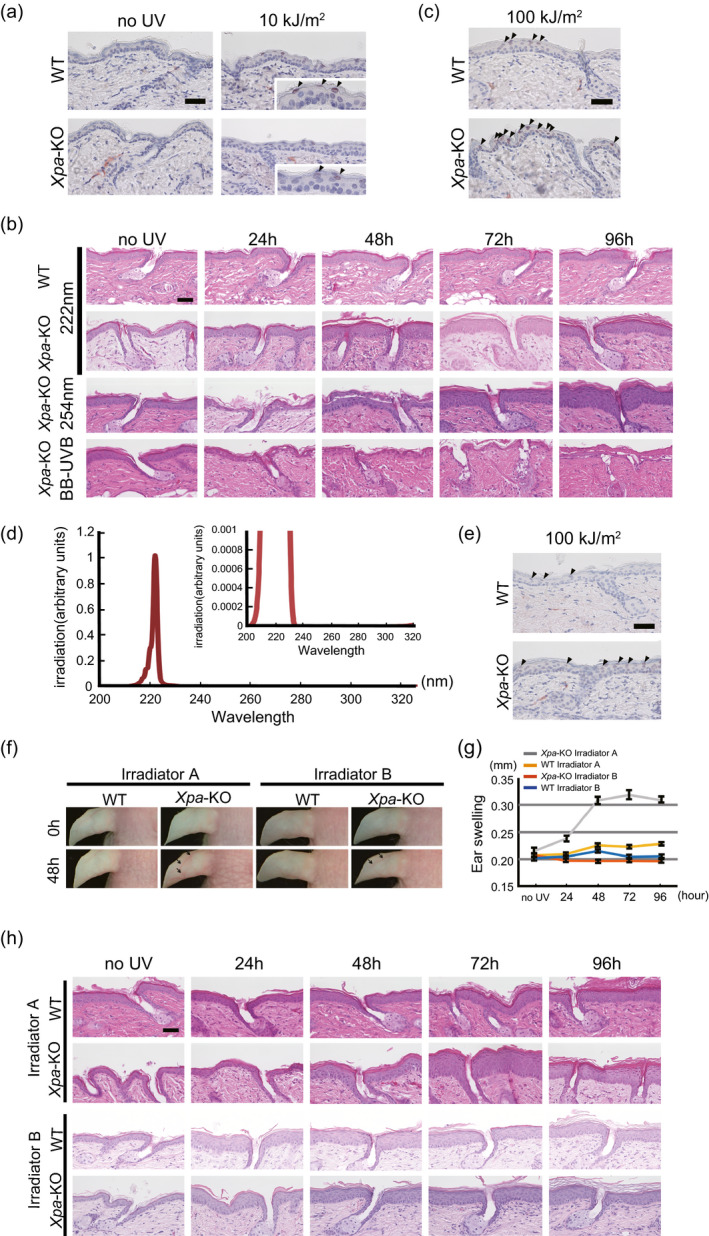
222‐nm UVC itself does not produce CPDs and inflammatory responses in skin. (a) Dorsal skin of *Xpa*‐knockout mice and wild‐type mice was taken 3 h after 222‐nm UVC exposure with 10 kJ m^−2^ and subjected to immunohistochemistry to detect CPDs. Inset photographs were magnified at the epidermis where positively faint stained cells were indicated with arrowheads. Scale bar: 50 µm. (b) Skin at indicated times following UV exposure (10 kJ m^−2^ 222‐nm UVC, 5.0 kJ m^−2^ 254‐nm UVC and 1.0 kJ m^−2^ broad‐band UVB) stained with hematoxylin & eosin. Scale bar: 50 µm. (c) Immunohistochemical detection of CPDs in skin from *Xpa*‐knockout and wild‐type mice irradiated with 222‐nm UVC at a high dose of 100 kJ m^−2^, 3 h post‐exposure. Arrowheads indicate positive cells. Scale bar: 50 µm. (d) Spectra of irradiator B, showing reduction in intensity in 235–280 nm wavelengths UV to <1% of the dose from irradiator A. In the UVC range, the integrating intensity of 230–235 nm wavelengths UV is 0.209% of the integrating intensity of 200–230 nm wavelengths UV, the integrating intensity of 235–280 nm wavelengths UV is 0.001% of 200–230 nm wavelengths UV, and in the UVB region, the integrating intensity of 280–320 nm wavelengths UV is 0.004% of 200–230 nm wavelengths UV. (e) Immunohistochemical detection of CPDs in *Xpa*‐knockout mice and wild‐type mice irradiated with high dose, 100 kJ m^−2^ of 222‐nm UVC from irradiator B 3 h after exposure. Arrowheads indicate positive cells. Scale bar: 50 µm. (f) Erythema after exposure to irradiator A and irradiator B, with dilatation of vessels of the ears and scaly erythema of the heads of *Xpa*‐knockout mice. (g) Ear swelling measured after 222‐nm UVC irradiation from irradiator A or irradiator B. Error bars represent standard error. (h) Histology after irradiation with 100 kJ m^−2^ of 222‐nm UVC using irradiator A or irradiator B. Skin sections were stained with hematoxylin & eosin. Scale bar: 50 µm.

We had already identified CPDs‐positive cells in the outermost epidermal layer of both genotypes by irradiation with 5.0 kJ m^−2^ of 222‐nm UVC (Fig. 2). When both genotype mice were irradiated with doubled doses of 222‐nm UVC 10 kJ m^−2^, more clearly visible CPDs‐positive cells were confirmed in the outermost epidermal layer at 3 h postirradiation (Fig. 6a). Interestingly, in *Xpa*‐knockout mice, epidermal thickening was observed after irradiation at this dose, peaking at 72 h post‐UV exposure (Fig. 6b), that was not observed in wild‐type mice. Similarly, remarkable epidermal hyperplasia was recognized in *Xpa*‐knockout mice after 254‐nm UVC (Fig. 6b). Based on these results, we investigated whether formation of CPDs and significant epidermal thickening with this relatively higher dose of 222‐nm UVC was caused solely by the 222‐nm UVC itself or other small fractions between 235–280 nm wavelengths UV (Fig. 1). We set the dose of 222‐nm UVC to 100 kJ m^−2^ for evaluation of CPDs formation and epidermal thickening. As shown in Fig. 6c, abundant CPDs‐positive cells were observed in the outermost layer of epidermis, irrespective of genotype. We constructed a second 222‐nm UVC lamp unit, irradiator B, which was designed to reduce the amount of radiation dose in 235–280 nm wavelengths UV to <1% of that of irradiator A (Figs. 1 and 6d). When we reevaluated CPDs formation and epidermal thickening using the same dose with irradiator B, 100 kJ m^−2^, the number of CPDs‐positive cells was remarkably reduced (Fig. 6e) and significant epidermal thickening peaking at 72 h post‐UV exposure observed by irradiation with irradiator A was not observed in either genotype (Fig. 6h). However, slight epidermal hyperplasia was observed from 24 to 96 h post‐UV exposure with irradiator B in both genotypes (Fig. 6h). We reevaluated the inflammatory response with 222‐nm UVC, 100 kJ m^−2^ from irradiators A and B, comparing ear swelling and erythema. Vasodilatation and swelling of the ear were observed in *Xpa*‐knockout mice irradiated with irradiator A. When we used irradiator B, no ear swelling, but very slight vasodilatation of ears, was noted in the *Xpa*‐knockout mice. In wild‐type mice, no effects were observed with either irradiator (Fig. 6f,g). These results suggest that 222‐nm UVC itself is much less inflammatory.

## Discussion

Our results indicated that long‐term irradiation of mice with 222‐nm UVC failed to induce nonmelanoma skin tumors, even in *Xpa*‐knockout mice. Theoretically, there are two possible reasons for this. One is that efficacy for formation of CPDs, the main photo‐induced DNA lesion which is closely associated with photocarcinogenesis, is highest at approximately 260 nm and decreases at shorter and longer wavelengths, and generation of CPDs at 222 nm is approximately 70% of that at 254 nm, as previously shown in the action spectrum for the formation of CPDs (29). The second is that the 222‐nm UVC is too short to penetrate the stratum corneum to reach the basal cell layer, where cancer stem cells are considered to reside (30, 31). However, CPDs formation was observed in the outermost layer of epidermis in both genotypes of mice at the higher dose, 5.0 kJ m^−2^, which could be accentuated by an extremely high dose of 100 kJ m^−2^ (Figs. 2 and 6a,c). To identify whether it was due to the 222‐nm UVC itself or other small fractions irradiated from the 222‐nm UVC lamp, we measured the depth to which 222‐nm UVC can penetrate human corneal tissue. The spectral transmittance at 222 nm was measured using human stratum corneum, showing nearly zero penetrance (0.001%, see Figure S2). Based on previous reports showing that CPDs formation caused by 235–280 nm wavelengths UV was 10–100 times greater than that caused by 280–320 nm wavelengths UV (29), we postulated that a small fraction of longer‐wavelength UV from irradiator A could be causing the formation of CPDs. Therefore, we constructed irradiator B, which reduced the radiation dose in 235–280 nm wavelengths UV to <1% of that produced by irradiator A. The number of CPDs‐positive cells produced by irradiator B was remarkably reduced in both genotypes of mice compared to that produced by irradiator A (Fig. 6c,e). Taken together, formation of CPDs, yet low number of those, (Figs. 2 and 6a) by irradiator A with a higher dose than that used for germicidal purposes could be attributed to a small component of 235–280 nm wavelengths UV. Most importantly, however, we have shown that 222‐nm UVC lamps, irrespective of installation in irradiator B or irradiator A, at even extremely high doses could not reach the basal layer. This is important because CPDs harboring basal cells may sustain cancer‐prone mutations when they enter the cell cycle (32). CPDs containing epidermal cells in the outermost layer would be destined for the stratum corneum in a shorter time course, over a few days (33), at which time they would no longer be capable of transformation. In addition, since a large portion of 222‐nm UVC is absorbed by keratin in the stratum corneum, theoretically, only a small proportion of the initially incident 222‐nm UVC would reach the epidermal layer. In fact, *in vitro* study examining the penetration of 222‐nm UVC into artificial corneal tissue showed only < 0.001 % of initial light energy to have reached the epidermal cells (see Figure S2).

**Figure 7:**
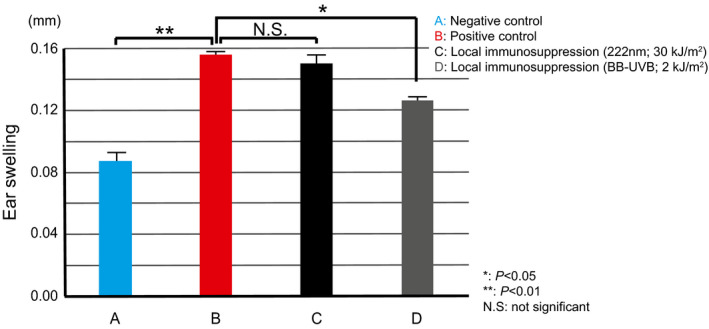
UV‐Induced Local Immunosuppression of CHS. For sensitization and elicitation of contact hypersensitivity (CHS), mice were sensitized by topical application of 100 µL of 7% 2,4,6‐trinitrochlorobenzene (TNCB) solution in acetone: olive oil (4:1) on abdominal skin (negative control: Bar A). CHS was elicited by application of 20 µL of a 1% TNCB solution on the surface of each side of both ears 6 days after sensitization. Ear thickness was measured before and 24 h after challenge, with the difference between the two readings recorded as the ear swelling (positive control: Bar B). To study UV‐induced local immunosuppression by 222‐nm UVC, the abdominal area of mice was irradiated with 2.0 kJ m^−2^ UVB (Bar D) or 30 kJ m^−2^ 222‐nm UVC (Bar C) on day 0, and sensitization was achieved at the irradiated site on day 3. Elicitation at both ears was performed on day 6. During UV irradiation, ears were protected from UV by adhesive tape, which was removed after exposure. There were no significant differences between mice irradiated with 222‐nm UVC before sensitization (Bar C) and those without UV irradiation (Bar B), while mice which received sensitization after broad‐band UVB presented with significantly reduced response (Bar D). *P < 0.05, **P < 0.01. Bars represent standard error.

The cutaneous inflammatory response caused by UV is a crucial factor in UV‐induced skin carcinogenesis. We previously showed that higher inflammatory responses are associated with higher tumor incidence in wild‐type mice (23) as well as DNA repair deficient mice including *Xpa*‐knockout and *Ogg1*‐knockout mice, both of which are deficient in removing dipyrimidine photoproducts and 8‐oxo‐7,8‐dihydroguanine (oxidatively generated DNA damage), respectively (21, 23, 34). In the present study, we showed that there was no elevation of CXCL1 along without ear swelling after 222‐nm UVC irradiation, which implies no carcinogenic promotion consequences of 222‐nm UVC property. On the other hand, we also must consider other immune‐mediators or immunomodulators in the context of UV‐induced skin carcinogenesis. Another important factor in immunomodulatory reactions in photocarcinogenesis is immunosuppression (35). Interleukin‐10 (IL‐10) is a potent immunosuppressive cytokine, produced by keratinocytes (36) following UVB exposure; enhanced UV‐induced immunosuppression has been reported in *Xpa*‐knockout mice (37). Furthermore, *Xpa*‐knockout mice are known to show strong enhanced immunosuppressive effects and inflammatory reactions following UVB exposure (38); these are thought to be associated with susceptibility to skin carcinogenesis (39). In order to clarify the possible effect of 222‐nm UVC on immune response, we investigated UV‐induced local immune suppression by means of contact hypersensitivity using 2,4,6‐trinitrochlorobenzene (TNCB) after 222‐nm UVC exposure and broad‐band UVB exposure as a positive control. No UV‐induced immunosuppressive effects were observed in *Xpa*‐knockout mice after 222‐nm UVC irradiation (Fig. 7), while suppression was observed after broad‐band UVB exposure. In a report from the 1960s, the required 222‐nm UVC dose to produce erythema in rabbit skin was estimated to be 0.5 kJ m^−2^ (40). That appears to have been an overestimate given our data showing no erythema at 10 kJ m^−2^ in *Xpa*‐knockout mice. Our data clearly indicate that even a relatively high dose of 222‐nm UVC irradiation does not induce either effect linked to skin tumor formation: inflammation and immunosuppression.

In conclusion, we suggest the safety of 222‐nm UVC lamps for the sterilization of human skin, with no evidence of skin carcinogenesis on long‐term exposure, even for highly skin photocarcinogenesis phenotype mice, in addition to no inflammatory reactions and no harmful effects on mouse eyes.

## Conflict of Interests

A.NS., H.O. and T.I. have the following competing interests: A.NS., H.O. and T.I. are provided support in the form of salaries from Ushio Inc., Tokyo, Japan. We declare no other employments, consultancy and patents of this funder, Ushio Inc. No further intellectual property relating to this paper will be developed.

## Supporting information


**Figure S1.** Chronic 222‐nm UVC does not cause skin tumors on *Xpa*‐knockout mice with multiple wounds.

**Figure S2.** The spectral transmittance at 222‐nm UVC of human stratum corneum.
