# Supplementary material for: Long‐term Effects of 222‐nm ultraviolet radiation C Sterilizing Lamps on Mice Susceptible to Ultraviolet Radiation
Source: Photochem Photobiol. 2020 May 31;96(4):853–62. doi: 10.1111/php.13269 (PMC7497027; doi:10.1111/php.13269)
Supplement: Supplementary file 1 — Figure S1. Chronic 222‐nm UVC does not cause skin tumors on Xpa‐knockout mice with multiple wounds. [file PHP-96-853-s001.pdf]

Supporting Information

Long-term effects of 222 nm ultraviolet radiation C sterilizing lamps on mice susceptible to ultraviolet radiation

Nozomi Yamano, Makoto Kunisada, Sachiko Kaidzu, Kazunobu Sugihara, Aiko Nishiaki-Sawada, Hiroyuki Ohashi, Ai Yoshioka, Tatsushi Igarashi, Akihiro Ohira, Masaki Tanito, and Chikako Nishigori

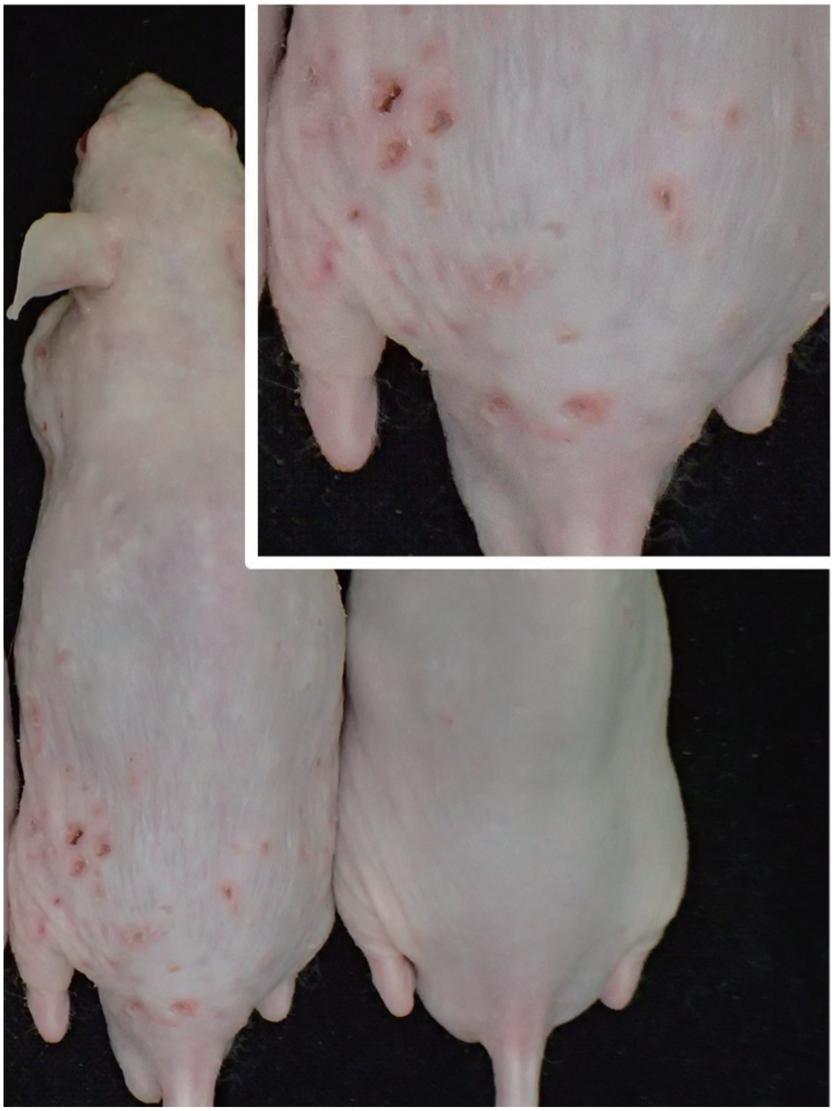

Figure S1. Chronic 222 nm-UVC does not cause skin tumors on *Xpa*-knockout mice with multiple wounds.

No tumor formation in *Xpa*-knockout male mice despite a number of wounds caused by fighting after irradiation as in Figure 4. Photograph in inset shows scratches and biting scars from fighting.

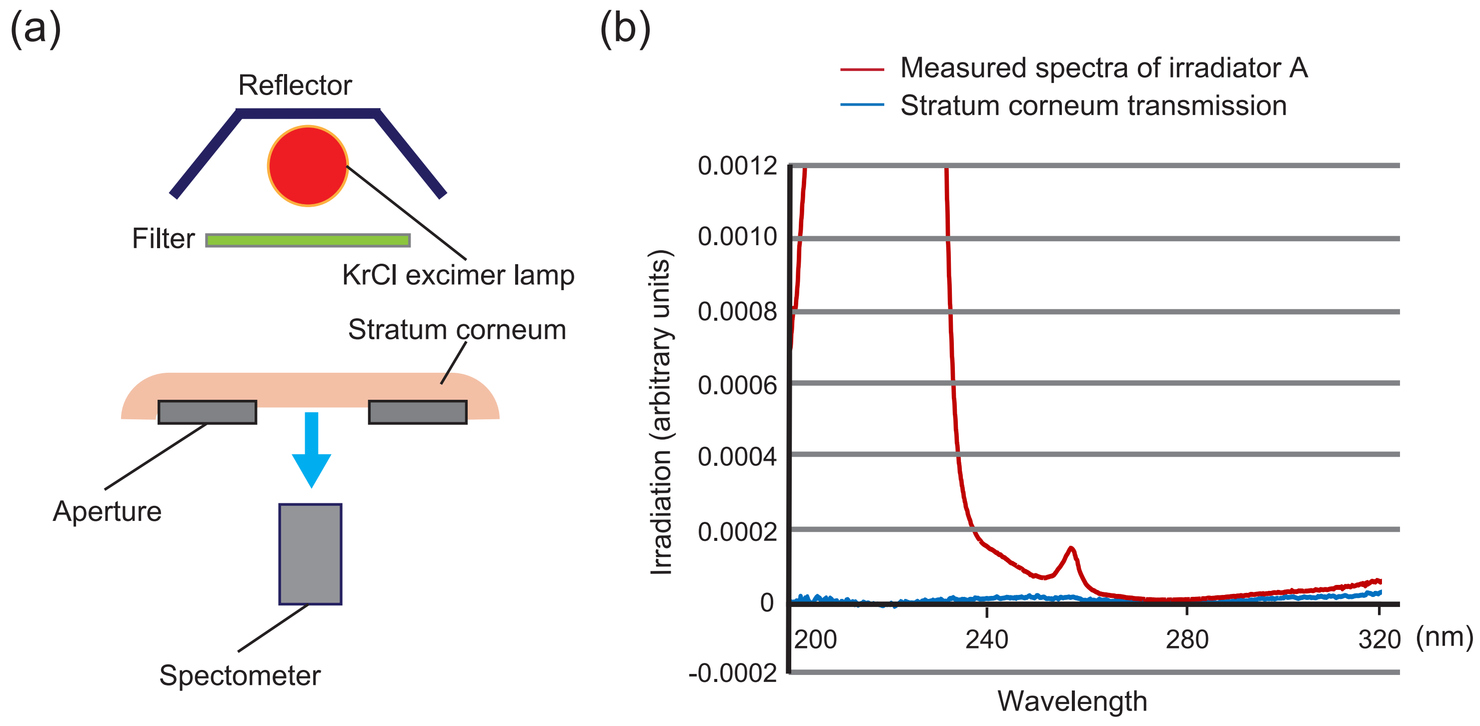

Figure S2. The spectral transmittance at 222 nm-UVC of human stratum corneum.

(a) Experimental apparatus for measurement of spectrum through the human primary stratum corneum transmission by irradiator A.

UV radiation from irradiator A penetrates a human primary stratum corneum (Biopredic International, France) through 6mm diameter aperture with the light was detected by a QE-Pro spectrometer (Ocean Optics, Largo, FL), resolution is 0.036nm and has a CC-3-UV-S diffuser.

(b) The red line is the spectrum in the absence of stratum corneum. The blue line is the spectrum after passing through the stratum corneum. The vertical axis was magnified 1,000-fold for ordinary arbitrary units. The intensity of 200-230 nm wavelengths UV after stratum corneum was reduced to 0.001%. Previous studies showed that wavelengths less than 230 nm do not penetrate the stratum corneum of the human skin (Ref.1-3)

1) Pathak, M.A and Epstein, J.H. (1971) Normal and abnormal reaction to light : *in* Dermatology in General medicine edited by Fitzpatrick, T.B, *et al.* McGraw Hill Book Company, New York, 977-1036.

2) van Gemert, M.J., Jacques, S. L., Sterenborg, H. J. and Star, W. M. (1989) Skin optics. *IEEE Trans Biomed Eng* **36**, 1146-1154.

3) Everett, M. A., E. Yeargers, R. M. Sayre and R. L. Olson (1966) Penetration of epidermis by ultraviolet rays. *Photochem Photobiol* **5**, 533-542.
